# Steady-state solutions for the Muskat problem

**DOI:** 10.1007/s13348-021-00348-z

**Published:** 2022-03-08

**Authors:** Omar Sánchez

**Affiliations:** Instituto de Ciencias Matemáticas, Universidad Autónoma de Madrid, Madrid, Spain

**Keywords:** Muskat problem, Fingering patterns, Steady state solutions, Periodic solution, 34A12, 34C25, 76D45, 76S05

## Abstract

In this paper we study the existence of stationary solutions for the Muskat problem with a large surface tension coefficient. Ehrnstrom, Escher and Matioc studied in Mats Ehrnström (Methods Appl Anal 20:33-46, 2013) that there exists solutions to this problem for surface tensions below a finite value. In these notes we go beyond this value considering large surface tension. Also by numerical simulation we show some examples that explains the behavior of solutions.

## Introduction

**Fig. 1:**
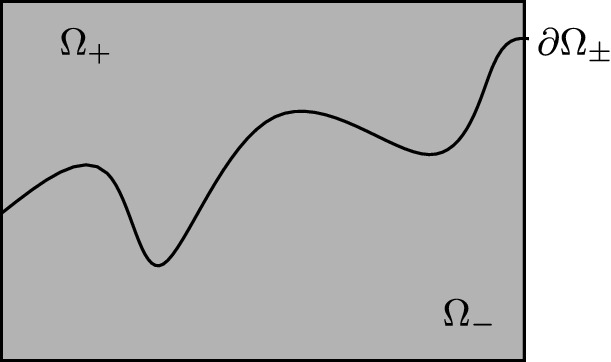
The interface

We consider two incompressible fluids with different densities and equal viscosity in a porous medium, separated by a curve $z=(z_1,z_2)$. The dynamic of this two phase-flow can be modelled by Incompressible Porous Media equation (IPM) which is given by the Darcy’s law, the mass conservation equation and the incompressibility condition

1 $$\begin{aligned} \begin{aligned}&\frac{\mu }{\kappa }v=-\nabla p-(0,\mathrm {g}\,\rho ),\\&\partial _t \rho +v\cdot \nabla \rho =0,\quad \, \\&\nabla \cdot v=0, \end{aligned} \end{aligned}$$

where *v* is the incompressible velocity, *p* is the pressure, $\mu$ is the dynamic viscosity, $\kappa$ is the permeability of the medium, $\rho$ is the liquid density and $\mathrm {g}$ is the acceleration due to gravity. Without loss of generality, we set $\mu /\kappa =1.$ The initial density, which takes only two constant values is defined by

$$\begin{aligned} \rho _0(x)=\left\{ \begin{array}{rl} \rho _+ &{}\, \text{ in } \, \varOmega _+(0),\\ \rho _- &{}\, \text{ in } \, \varOmega _-(0), \end{array}\right. \end{aligned}$$

for $x=(x_1,x_2)\in {\mathbb {R}}^2$ (see Fig. 1).

The research on the Muskat problem has been very intense due to the many applications (see [16, 20, 23] and [18]), one of the characteristic of this problem is that some scenarios are unstable and leads to an ill-posed problem, this scenario is when the heavier fluid is above the other one. In the stable scenario when the heavier fluid is in the lower part, the problem is locally well-posed. This arises from the linear stability analysis of the equation for the interface evolution (see [2–4] and [22]). Such linear stability is characterized by the sign of the Rayleigh–Taylor function

$$\begin{aligned} \sigma =(\rho _--\rho _+)\partial _\alpha z_1, \end{aligned}$$

for a curve $z=(z_1,z_2)$ that parameterizes the interface between the fluids. In the stable case as mathematical case is a challenging example of a free boundary problem driven by a nonlinear and non-local parabolic equation. In the unstable case recent investigations have show that the method of convex integration yields infinitely many weak solutions (see [5] and [6]). There is little doubt that the most important open questions is to understand a selection principle for such weak solutions. Recently the case of stationary IPM has been studied from this perspective in [15] by Hitruhin and Lindberg.

If we consider surface tension, there is a jump discontinuity of the pressure across the interface which is modeled to be equal to the local curvature times the surface tension coefficient

$$\begin{aligned} p_+-p_-=\gamma \,k(z). \end{aligned}$$

In this case we have viscous fingering, a phenomenon known in porous media as a result of an instability of the interface when a lower density fluid penetrates a more viscous fluid, the result is the formation of patterns of type finger in the interface. The free boundary problem is well posed (see [7] and [11]), since capillarity eliminates instabilities in Rayleigh-Taylor condition and this problem has been studied extensively (see [1, 10, 14, 17, 21] and [13]). For further results on the Muskat problem with and without surface tension see the survey [12].

In this work we give a description of the stationary solutions for the Muskat problem with surface tension, looking for $2\pi$ periodic solutions, this can be achieved by reducing the Muskat equation (2) to an ordinary differential equation (5). Using the Darcy’s Law we compute the vorticity

$$\begin{aligned} \nabla ^\perp \cdot v=\Big [\gamma \, \partial _\alpha k(z)(\alpha )+\mathrm {g}\,(\rho _+-\rho _-) \partial _\alpha z_2(\alpha )\Big ]\delta (x-z). \end{aligned}$$

The Muskat equation is given by

2 $$\begin{aligned} \begin{aligned}&z_t(\alpha ,t)=\frac{1}{2\pi }PV\int _{{\mathbb {R}}}\frac{(z(\alpha ,t)-z(\beta ,t))^\perp }{|z(\alpha )-z(\beta ,t)|^2}\partial _\beta \Big [\gamma \kappa (z(\beta ,t))+\mathrm {g}\,(\rho _+-\rho _-)z_2(\beta ,t)\Big ]d\beta ,\\&z(\alpha ,0)=z_0(\alpha ). \end{aligned} \end{aligned}$$

Therefore steady-state solutions of (2) are solutions of the equation

3 $$\begin{aligned} \partial _\alpha \Big [\gamma \kappa (z)(\alpha )+\mathrm {g}(\rho _+-\rho _-) z_2(\alpha )\Big ]=0, \end{aligned}$$

and a solution of (3) is a steady-state solutions of (2) and therefore a steady-state solutions of the Muskat problem. In this work we take by simplicity equal viscosities, if we consider different viscosities the stationary equation obtained is the same as in the case of equal viscosities. For a treatment of the case with different viscosities see [19], Sect. 6. From here, a curve $z:{\mathbb {R}}\rightarrow {\mathbb {R}}^2$ is a steady-state solution of (2) if satisfies

$$\begin{aligned} \gamma \frac{z_1'z_2''-z_1''z_2'}{\left( z_1'^2+z_2'^2\right) ^{3/2}}+\mathrm {g}\,\left( \rho _+-\rho _-\right) z_2=const\quad \hbox { and } \quad \int _{{\mathbb {S}}}z_2dx=0, \end{aligned}$$

where we consider that $z_2,$ the second component of the curve, is a odd function. Ehrnstrom, Escher and Matioc in [8] found a finite limit value with the property that if the surface tension coefficient remains below this value the curve remains $2\pi$ periodic and when the surface tension coefficient approaches to this finite value from below, the maximal slope of the curve tends to infinity.

The equation (3) in the stable case $\rho _->\rho _+$ has only trivial solution (see [9]). In this notes we consider the unstable case, when $\rho _+>\rho _-,$ that is, when the heavier fluid occupies the upper part and we are interesting in describing a solution with the following initial data

4 $$\begin{aligned} z(0)=(0,0)\quad \hbox { and }\quad z'(0)=(-\alpha ,1),\quad \alpha >0. \end{aligned}$$

We take

$$\begin{aligned} \lambda =\frac{\mathrm {g}(\rho _+-\rho _-)}{\gamma }, \end{aligned}$$

and recall the definition of the beta function

$$\begin{aligned} B(x,y)=\int _{0}^1 t^{x-1}(1-t)^{y-1}dt, \quad \mathrm {Re}(x),\mathrm {Re}(y)>0. \end{aligned}$$

The main result of this notes is the following theorem:

### Theorem 1

Given

$$\begin{aligned} \lambda _*=\frac{1}{2\pi ^2}B^2\left( \frac{3}{4},\frac{1}{2}\right) , \end{aligned}$$

there exists $\lambda ^*>0$ greater than $\lambda _*$ such that for each $\lambda \in (\lambda ^*,\lambda _*]$ there exists a unique $\alpha (\lambda )\in [0,\infty )$ and a periodic solution curve of the steady-state equation (3) that does not self-intersect.

In [8], Ehrnstrom, Escher and Matioc consider the parameter $\lambda >\lambda _*$ and in this case the curve is locally the graph of a function (*x*, *f*(*x*)),  instead of that we consider a curve (*g*(*y*), *y*) and we are able to show that there exists $2\pi$ periodic solutions for the case $\lambda ^*<\lambda <\lambda _*,$ moreover we show that if $\lambda <\lambda ^*$ the solutions there are no longer periodic. The proof in the main theorem is obtained by analyzing a explicit formula (7) for the period. Moreover we describe some numerical examples that indicates $\lambda ^*\sim \lambda _*/7.$

## Steady-state solutions

In this section we study the period of the steady-state solution with the conditions (4), under such conditions the curve *z* is a graph respect to a function *h*,  that is, $z(y)=(h(y),y)$ and the curve is a steady-state solution if the function *h* satisfies

5 $$\begin{aligned} - \frac{h''}{(1+h'^2)^{3/2}}+\lambda y=0,\quad h(0)=0,\quad h'(0)=-\alpha <0. \end{aligned}$$

To prove the main theorem we have three previous lemmas, the first about the existence of $2\pi$ periodic solutions, the second related to the function $\lambda \mapsto \alpha$ and the third on the intersection of the solution curves. We leave at the end of the section the proof of the main theorem.

The idea is integrate the equation (5) directly over the interval [0, *y*] to determine conditions in the parameters $\lambda$ and $\alpha .$ After the integration we have the next equation

$$\begin{aligned} \frac{h'(y)}{\left( 1+h'(y)^2\right) ^{1/2}}=\frac{\lambda }{2}y^2-\frac{\alpha }{\left( 1+\alpha ^2\right) ^{1/2}}. \end{aligned}$$

In order to simplify the notations we put $\displaystyle {\beta :=\frac{\alpha }{(1+\alpha ^2)^{1/2}}.}$ Taking squares and since $h'$ and $(\lambda /2)y^2-\beta$ has the same sign, we have

6 $$\begin{aligned} h(y)=\int _{0}^y\frac{\left( \frac{\lambda }{2}s^2-\beta \right) }{\sqrt{1-\left( \frac{\lambda }{2}s^2-\beta \right) ^2}}ds. \end{aligned}$$

We observe from equation (6) that there exists a zero for $h'$ and a positive value where $h'$ explodes, that is

$$\begin{aligned} h'\left( \sqrt{\frac{2}{\lambda }\beta }\right) =0 \quad \hbox { and }\quad h'\left( \sqrt{\frac{2}{\lambda }(1+\beta )}\right) =\infty , \end{aligned}$$

also at this point the curve is no longer the graph of the function *h*. We define the period of the solution curve as the integral

7 $$\begin{aligned} T(\lambda ,\alpha ):=\int _{0}^{\sqrt{2\lambda ^{-1}(1+\beta )} } \frac{\left( \frac{\lambda }{2}s^2-\beta \right) }{\sqrt{1-\left( \frac{\lambda }{2}s^2-\beta \right) ^2}} ds. \end{aligned}$$

### Lemma 1

If $\lambda \in ( 0,\lambda _*],$ the period (7) of the solution curve satisfies

$$\begin{aligned} T(\lambda ,\infty )<\pi /2\le T(\lambda , 0). \end{aligned}$$

### Proof

Taking the change of variable $\tau =s/\sqrt{2\lambda ^{-1}(1+\beta )}$, we get the next expression for the period

8 $$\begin{aligned} T(\lambda ,\alpha )=\sqrt{\frac{2}{\lambda }}\int _{0}^{1}\frac{g_\tau (\alpha )}{\sqrt{1+g_\tau (\alpha )}}\frac{d\tau }{\sqrt{1-\tau ^2}}, \end{aligned}$$

where $g_\tau (\alpha )=(1+\beta )\tau ^2-\beta .$ The derivatives respect to $\beta$ and $\alpha$ of $g_\tau$ and $\beta$ respectively are

$$\begin{aligned} \frac{\partial }{\partial \beta }g_\tau (\alpha )=\tau ^2-1<0\quad \hbox { and }\quad \frac{\partial \beta }{\partial \alpha }=\frac{1}{(1+\alpha ^2)^{3/2}}>0. \end{aligned}$$

Then from the chain rule the derivative respect to $\alpha$ is

$$\begin{aligned} g_\tau '(\alpha )=\frac{\partial }{\partial \beta }g_\tau (\alpha )\frac{\partial \beta }{\partial \alpha } =\frac{\tau ^2-1}{(1+\alpha ^2)^{3/2}}<0 \end{aligned}$$

hence

9 $$\begin{aligned} \frac{d}{d\alpha }\left( \frac{g_\tau (\alpha )}{\sqrt{1+g_t(\alpha )}}\right) =g_\tau '(\alpha )\frac{2+g_\tau (\alpha )}{(1+g_\tau (\alpha ))^{3/2}}<0 \end{aligned}$$

and therefore the period $T(\lambda ,\alpha )$ is decreasing with respect to $\alpha .$ Now, if we want to determine the pair $(\lambda ,\alpha )$ such that $T(\lambda ,\alpha )=\pi /2,$ we will determine conditions on $\lambda$ observing its behavior as $\alpha$ goes to zero and infinity. We will see explicitly that the following inequalities are satisfied

$$\begin{aligned} \lim _{\alpha \rightarrow \infty }T(\lambda ,\alpha )<\pi /2 <\lim _{\alpha \rightarrow 0}T(\lambda ,\alpha ). \end{aligned}$$

For the first inequality, we compute $T(\lambda ,0)$ from (8), that is

$$\begin{aligned} \lim _{\alpha \rightarrow 0}T(\lambda ,\alpha )=\int _{0}^{1}\frac{g_\tau (0)}{\sqrt{1+g_\tau (0)}}\frac{d\tau }{\sqrt{1-\tau ^2}}. \end{aligned}$$

When $\alpha =0,$ $\beta = 0$ and we have

10 $$\begin{aligned} T(\lambda ,0)=\frac{1}{2}\frac{1}{\sqrt{2\lambda }}\int _{0}^{1}\frac{\tau ^{-1/4}}{(1-\tau )^{1/2}}d\tau =\frac{1}{2}\frac{1}{\sqrt{2\lambda }}\int _{0}^{1}\tau ^{3/4-1}(1-\tau )^{1/2-1}d\tau . \end{aligned}$$

Therefore, if we take

$$\begin{aligned} \lambda <\frac{1}{2\pi ^2}B^2\left( \frac{3}{4},\frac{1}{2}\right) =\lambda _*, \end{aligned}$$

we get

$$\begin{aligned} T(\lambda ,0)=\frac{1}{2}\frac{1}{\sqrt{2\lambda }}B\left( \frac{3}{4},\frac{1}{2}\right) >\frac{\pi }{2}. \end{aligned}$$

The next step is compute the limit when $\alpha \rightarrow \infty$ in equation (7). We observe for a fixed $\lambda <\lambda _*$ the limit in the integral satisfies

11 $$\begin{aligned} \begin{aligned} \lim _{\alpha \rightarrow \infty }T(\lambda ,\alpha )&=\lim _{\alpha \rightarrow \infty }\sqrt{\frac{2}{\lambda }}\int _{0}^{1}\frac{g_\tau (\alpha )}{\sqrt{1+g_\tau (\alpha )}}\frac{d\tau }{\sqrt{1-\tau ^2}}\\&=\sqrt{\frac{2}{\lambda }}\left( \int _0^{1/\sqrt{2}}+\int _{1/\sqrt{2}}^1\right) \frac{g_\tau (\infty )}{\sqrt{1+g_\tau (\infty )}}\frac{d\tau }{\sqrt{1-\tau ^2}}\\&=\sqrt{\frac{2}{\lambda }}\int _0^{1/\sqrt{2}}\frac{g_\tau (\infty )}{\sqrt{1+g_\tau (\infty )}}\frac{d\tau }{\sqrt{1-\tau ^2}}+ \frac{1}{\sqrt{\lambda }}\left( \sqrt{2}+\log \left( \frac{\sqrt{2}}{2+\sqrt{2}}\right) \right) . \end{aligned} \end{aligned}$$

We can see in the last integral, that for a fixed $\lambda <\lambda _*$

$$\begin{aligned} \lim _{\alpha \rightarrow \infty }T(\lambda ,\alpha )<0 \end{aligned}$$

moreover $T(\lambda ,\alpha )\rightarrow -\infty ,$ because the first integral goes to $-\infty .$ $\square$

The value $\lambda _*$ is the critical value found by Ehrnstrom, Escher and Matioc. When the parameter $\lambda \in (\lambda _*,1]$ the steady solution correspond to a curve parameterized by the graph (*x*, *f*(*x*)) with $f(0)=0$ and $f'(0)=\alpha$ (see [8]). The next lemma provides a relation between $\lambda$ and $\alpha .$

### Lemma 2

Let $\lambda \in (0,\lambda _*],$ then there exists a unique $\alpha (\lambda )\in [0,\infty )$ such that $T(\lambda ,\alpha (\lambda ))=\pi /2$ and the mapping $\alpha :(0,\lambda _*]\rightarrow [0,\infty )$ is smooth, bijective and decreasing.

### Proof

From lemma 1 we have $T(\lambda ,\infty )<\pi /2\le T(\lambda , 0)$ for $\lambda \in (0,\lambda _*].$ Also we known that $T(\lambda ,\alpha )$ is smooth respect to $\lambda$ and $\alpha ,$ hence for $\lambda \in (0,\lambda _*]$ there exists a unique $\alpha (\lambda )\in (0,\infty )$ such that $T(\lambda ,\alpha (\lambda ))=\pi /2.$ Choose the pair $(\lambda ,\alpha (\lambda ))\in (0,\lambda _*]\times [0,\infty )$ such that $T(\lambda ,\alpha (\lambda ))=\pi /2,$ then

$$\begin{aligned} 0=\frac{d}{d\lambda }T(\lambda ,\alpha (\lambda ))=\partial _\lambda T(\lambda ,\alpha (\lambda ))+\partial _\alpha T(\lambda ,\alpha (\lambda ))\,\alpha '(\lambda ), \end{aligned}$$

because $\partial _\alpha T,\partial _\lambda T<0$ we have $\alpha '(\lambda )<0.$ From (10) we get $\lim _{\lambda \rightarrow \lambda _*}\alpha (\lambda )=0,$ and from (11), $\lim _{\lambda \rightarrow 0}\alpha (\lambda )=\infty .$ This complete the proof. $\square$

To complete the proof of the main theorem we need to know if the curves solutions has intersections. We know from (7) that the function *h* has a minimum at the point $y=\sqrt{2\lambda ^{-1}\beta },$ define

$$\begin{aligned} I(\lambda ,\alpha ):=\int _{0}^{\sqrt{2\lambda ^{-1}\beta }} \frac{\left( \frac{\lambda }{2}y^2-\beta \right) }{\sqrt{1-\left( \frac{\lambda }{2}y^2-\beta \right) ^2}}dy \end{aligned}$$

which is the value of *h* at $\sqrt{2\lambda ^{-1}\beta }.$ Now we want to determine if the fact that $\lambda \in (0,\lambda _*]$ is enough to have

$$\begin{aligned} I(\lambda ,\alpha (\lambda ))>-\frac{\pi }{2}, \end{aligned}$$

this property is important because we want that the solution curve has not intersections. Taking the change of variable $\tau =s/\sqrt{2\lambda ^{-1}\beta }$ we can rewrite the integral as

$$\begin{aligned} I(\lambda ,\alpha )=\sqrt{\frac{2}{\lambda }}\int _{0}^1\frac{\beta (\tau ^2-1)}{\sqrt{1-\beta ^2(1-\tau ^2)^2}}\beta ^{1/2}d\tau , \end{aligned}$$

we have the next lemma.

### Lemma 3

There exists $\lambda ^*<\lambda _*$ positive such that if $\lambda \in (\lambda ^*,\lambda _*]$ then $I(\lambda ,\alpha (\lambda ))>-\pi /2$ and $I(\lambda ^*,\alpha (\lambda ^*))=-\pi /2$ .

### Proof

We observe

$$\begin{aligned} \partial _\lambda I(\lambda ,\alpha )=\int _{0}^1\frac{\beta (\tau ^2-1)}{\sqrt{1-\beta ^2(1-\tau ^2)^2}}\beta ^{1/2}d\tau \left( -\frac{1}{\sqrt{2}}\lambda ^{-3/2}\right) >0 \end{aligned}$$

because $0<\tau <1,$ then $I(\lambda ,\alpha )$ is increasing with respect to $\lambda .$ Take the pair $(\lambda ,\alpha (\lambda ))\in \mathbb (0,\lambda _*]\times [0,\infty ),$ such that $T(\lambda ,\alpha (\lambda ))=\pi /2.$ Now we have

$$\begin{aligned} \frac{\pi }{2}=I(\lambda ,\alpha (\lambda ))+\sqrt{\frac{2}{\lambda }}\int _{\sqrt{\frac{\beta }{\beta +1}}}^1\frac{g_{\tau }(\alpha )}{\sqrt{1+g_\tau (\alpha )}}\frac{d\tau }{\sqrt{1-\tau ^2}}, \end{aligned}$$

then we have

$$\begin{aligned} \begin{aligned}\lim _{\lambda \rightarrow \lambda _*}I(\lambda )&=\frac{\pi }{2}-\lim _{\lambda \rightarrow \lambda _*}\sqrt{\frac{2}{\lambda }}\int _{\sqrt{\frac{\beta }{\beta +1}}}^1\frac{g_\tau (\alpha )}{\sqrt{1+g_\tau (\alpha )}}\frac{d\tau }{\sqrt{1-\tau ^2}}\\&=\frac{\pi }{2}-\sqrt{\frac{2}{\lambda _*}}\int _{0}^{1}\frac{g_\tau (0)}{\sqrt{1+g_\tau (0)}}\frac{d\tau }{\sqrt{1-\tau ^2}}\\&=\frac{\pi }{2}-\frac{\pi }{2}=0, \end{aligned} \end{aligned}$$

the last inequality is due to $\lim _{\lambda \rightarrow \lambda _*}\alpha (\lambda )=0.$ Hence from $\lim _{\lambda \rightarrow 0}\alpha (\lambda ) =\infty ,$ we get

$$\begin{aligned} \begin{aligned}\lim _{\lambda \rightarrow 0} I(\lambda )&=\frac{\pi }{2}-\lim _{\lambda \rightarrow 0}\sqrt{\frac{2}{\lambda }}\int _{\sqrt{\frac{\beta }{\beta +1}}}^1\frac{g_\tau (\alpha )}{\sqrt{1+g_\tau (\alpha )}}\frac{d\tau }{\sqrt{1-\tau ^2}}\\&=\frac{\pi }{2}-\left( \lim _{\lambda \rightarrow 0}\sqrt{\frac{2}{\lambda }}\right) \left( \lim _{\alpha \rightarrow \infty }\int _{\sqrt{\frac{\beta }{\beta +1}}}^1\frac{g_\tau (\alpha )}{\sqrt{1+g_\tau (\alpha )}}\frac{d\tau }{\sqrt{1-\tau ^2}}\right) \\&=\frac{\pi }{2}-\left( \lim _{\lambda \rightarrow 0}\sqrt{\frac{2}{\lambda }}\right) \left( \sqrt{2}+\log \left( \frac{\sqrt{2}}{2+\sqrt{2}}\right) \right) =-\infty \end{aligned} \end{aligned}$$

therefore with respect to $\lambda$ we have

$$\begin{aligned} I(0)<-\frac{\pi }{2}<I(\lambda _*). \end{aligned}$$

The continuity of *I* over $(0,\lambda _*]$ implies that there exists $\lambda ^*>0$ such that $I(\lambda ^*)=-\pi /2.$ We take $\lambda \in (\lambda ^*,\lambda _*]$ then by lemma 1, there exists $\alpha (\lambda )\in [0,\infty )$ such that $T(\lambda ,\alpha (\lambda ))=\pi /2.$ Since $I(\lambda )$ is increasing with respect to $\lambda$ and $I(\lambda ^*)=-\pi /2$ we have

$$\begin{aligned} -I(\lambda ^*)<I(\lambda )<I(\lambda _*), \end{aligned}$$

which means that

$$\begin{aligned} I(\lambda )>-\frac{\pi }{2}. \end{aligned}$$

$$\square $$

Now we can proceed to the proof of the main result 1.

### Proof

***[of the main theorem*** ***1******]*** Take $\lambda \in (0,\lambda _*],$ then from lemmas 1 and 2 we find $\alpha (\lambda )$ such that $T(\lambda )=\pi /2.$ Now if $\lambda >\lambda ^*$ by lemma 3 we have value $I(\lambda )>-\pi /2.$ Hence by reflections we can construct a curve $2\pi$ periodic which does not have self intersections and is solution of the steady equation (3). $\square$

**Fig. 2:**
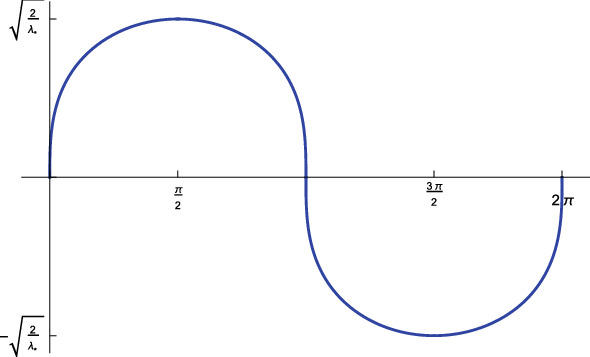
Curve solution for $\lambda _*$ and $\beta =0.$

## Numerical examples

**Fig. 3:**
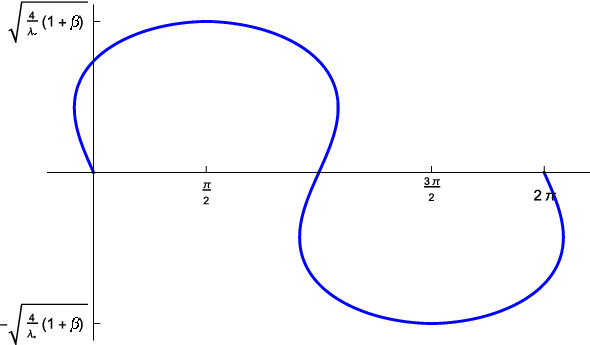
Curve solution for $\lambda _*/2$ and $\beta \sim 0.225$

**Fig. 4:**
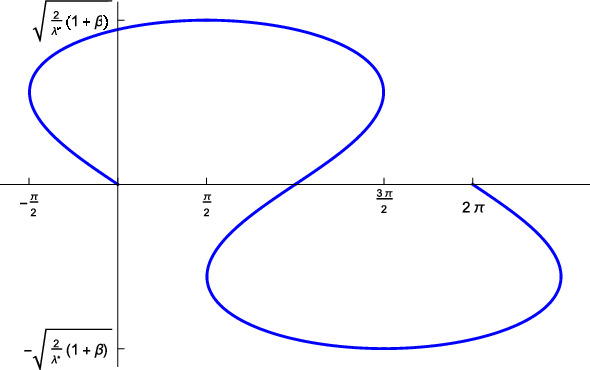
Curve solution for $\lambda ^*\sim \lambda _*/7$ and $\beta \sim 0.46$

**Fig. 5:**
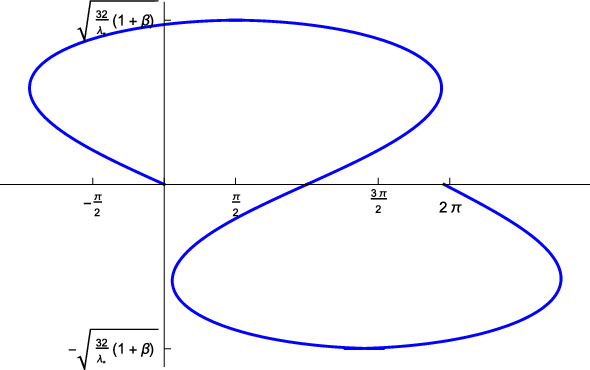
Curve solution for $\lambda _*/16$ and $\beta \sim 0.525$

In this section we show some numerical examples of steady-state solutions for different values of $\lambda .$ The Figs. 2, 3, 4 and 5 explains the behavior of the solution curves when $\lambda$ approaches to the value $\lambda ^*$. The limit curve $z_{\lambda ^*}$ (Fig. 4) remains $2\pi$ periodic but has self-intersections, also for $\lambda _*/16$ (Fig. 5) we observe that the curve solution has self-intersections, then we can infer that $\lambda _*/16<\lambda ^*.$

### Remark 1

It remains to have a analytical proof of the value $\lambda ^*$ that lead us to an explicit value for $\lambda ^*.$
